# Dataset for reproducing absorption spectra of methyl orange from the RGB values of microscopic images

**DOI:** 10.1016/j.dib.2020.105998

**Published:** 2020-07-05

**Authors:** Arinori Inagawa, Kana Saito, Asuka Sasaki, Nobuo Uehara

**Affiliations:** Graduate School of Regional Development and Creativity, Utsunomiya University, 7-1-2, Yoto, Utsunomiya, Tochigi 321-8585, Japan

**Keywords:** Absorption spectra, RGB, Microscopic images, Reproduction, Methyl orange

## Abstract

The present dataset is related to the research paper entitled “Reproducing Absorption Spectra of pH Indicators from RGB Values of Microscopic Images” (Inagawa et al., 2020). The dataset contains microscopic images of aqueous methyl orange (MO), absorption spectra acquired with a spectrophotometer, loading spectra and calculation sheets for reproducing absorption spectra of aqueous MO from their RGB values of the microscopic images. The microscopic transmission images of the standard MO solutions at various pH conditions were acquired with a CMOS camera equipped with an invert microscope. Meanwhile, the loading spectra were obtained by principle component analysis of a series of absorption spectra of the standard solutions. The conversion matrix from RGB values in a region of interests (ROI) to score values were linear-algebraically determined from the RGB values and score values of the standard solutions. The absorption spectra of the sample solutions of which pH conditions are unknown were then reproduced by calculating the linear combination of the loading spectra with the score values obtained from the conversion process. Herein, the absorption spectra of MO are reproduced at various pH and ROI conditions.

**Specifications Table**

**Table:** 

**Subject**	Analytical Chemistry
**Specific subject area**	Chemometrics and Microspectroscopy
**Type of data**	Images Figures
**How data were acquired**	An invert microscope (Diaphoto, Nikon, Japan), a white LED light source (MIC-209, AmScope, USA) and a CMOS camera (Model A35140U3, OMAX Microscope, USA) for acquisition of microscopic transmission images of pH indicator solutions A spectrophotometer (V-750, JASCO, Japan) equipped with plastic-made cuvettes with a light path length of 1 cm to measure absorption spectra of the sample solutions. Image analysis shareware ImageJ (NIH, USA) to obtain RGB values in the region of interests (ROI) in the acquired images. Data analysis shareware KyPlot5.0 (KyensLab Inc., Japan) for principle component analysis (PCA) of the spectrophotometric absorption spectra to obtain the loading spectra.
**Data format**	Raw: microscopic images and a set of spectrophotometric spectra Analyzed: loading spectra obtained by PCA and reproduced spectra.
**Parameters for data collection**	The pH of aqueous MO adjusted with acetic buffer. The ROI where the RGB values were analyzed.
**Description of data collection**	The sample solutions were transferred into a glass-sealed plastic cuvette. The microscopic images were acquired with the CMOS camera equipped with the invert microscope with white light irradiated by the LED light source attached above the sample. Absorption spectra of the sample solutions were measured with a spectrophotometer against water as references. A series of the spectra were analyzed by least square PCA with the data analysis shareware. The converting calculation from RGB values to absorption spectra were conducted on a Microsoft Excel Sheet 2016.
**Data source location**	Graduate School of Regional Development and Creativity, Utsunomiya University, 7–1–2, Yoto, Utsunomiya, Tochigi 321–8585, Japan
**Data accessibility**	Data are available in this article.
**Related research article**	A. Inagawa, A, Sasaki, N. Uehara, Reproducing Absorption Spectra of pH Indicators from RGB Values of Microscopic Images, *Talanta*, 216 (2020), 120,952. https://doi.org/10.1016/j.talanta.2020.120952

## Value of the data

• The absorption spectra of aqueous methyl orange (MO) were reproduced from RGB values of their microscopic images.
• Our dataset can provide proper calculation steps to reproduce chemical spectra from RGB values for those who are eager to obtain the absorption spectra in relatively small spaces in a short time.
• Reproducing absorption spectra of MO from the RGB values at a glance enables the monitoring pH values in small spaces in several milliseconds, which enables monitoring the pH values in cells, organs and other confined spaces continuously.
• The present dataset would also facilitate establishment of precise ratiometric detection systems with affordable CMOS sensors such as video cameras and smartphone, which promotes automated simple spectrophotometric detections.

## 1 Data description

The present dataset in this paper describes the spectrum reproduction procedure of aqueous methyl orange (MO) from RGB values of their microscopic images, which supports the applicability of our previous paper entitled “Reproducing absorption spectra of pH indicators from RGB values of microscopic images” [1]. All the data are preserved in the zip file as a supplementary material.

### 1.1 Acquisition of microscopic images and absorption spectra, and data analysis

**Fig. 1:**
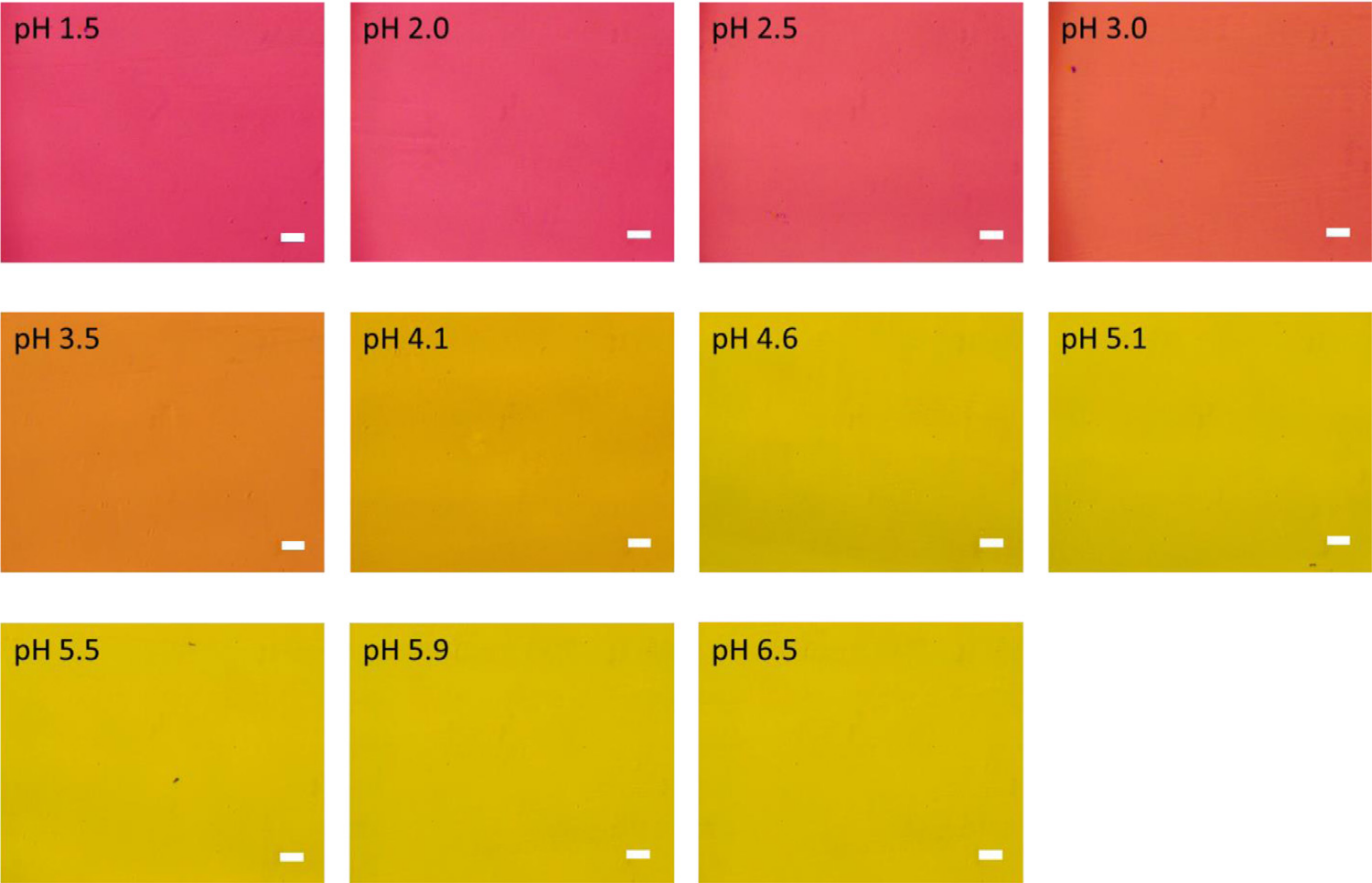
Microscopic images of aqueous MO at various pH conditions as standard solutions. The concentration of MO was set to 1.0 × 10^−5^ mol dm^−3^. The original data are preserved in a zip file as a supplementary material. Scale bars indicate 100 μm for every images.

**Fig. 2:**
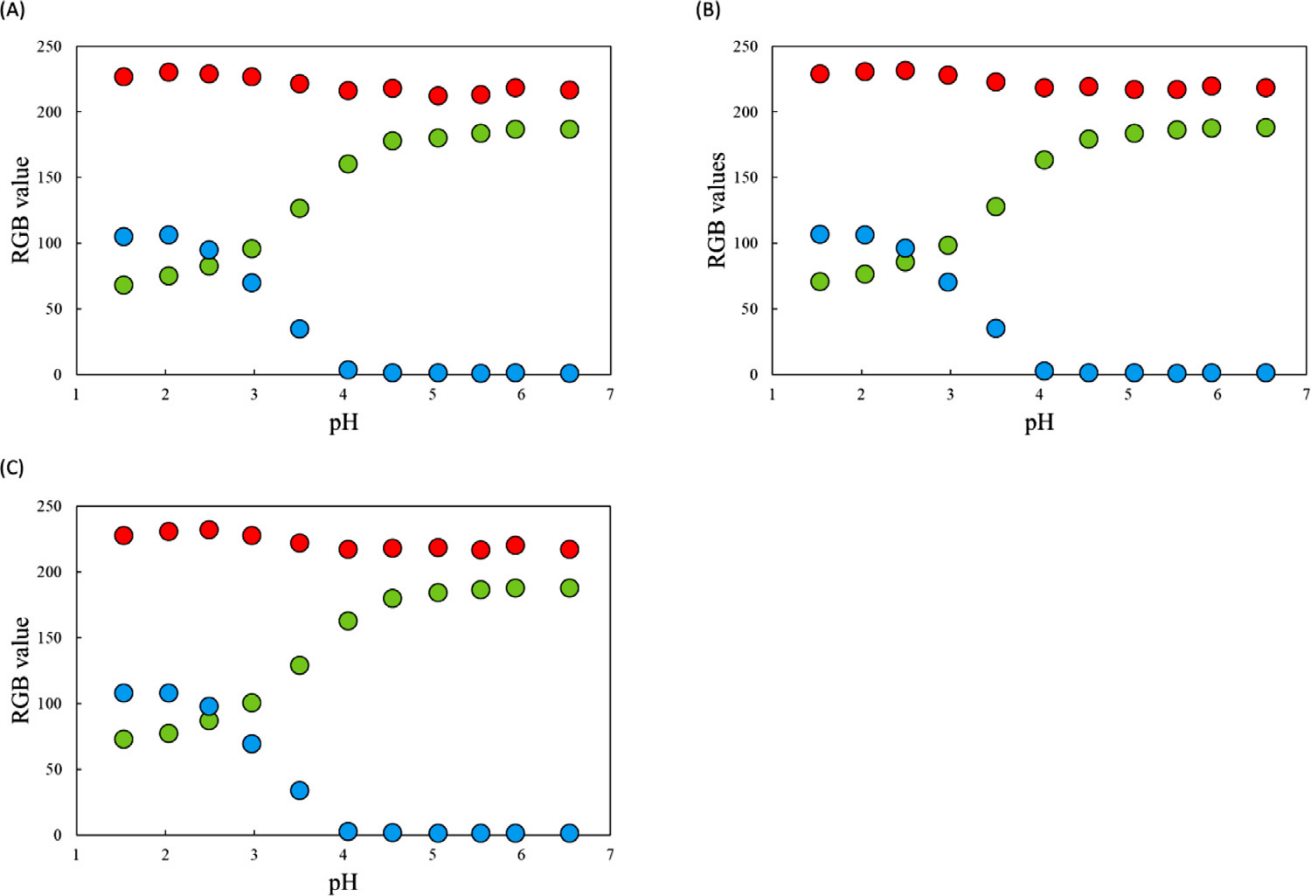
RGB values of the ROI in Fig. 1. The ROI was set to (A)1024 × 822 pixels (B)100 × 100 pixels, and (c) 10 × 10 pixels.

**Fig. 3:**
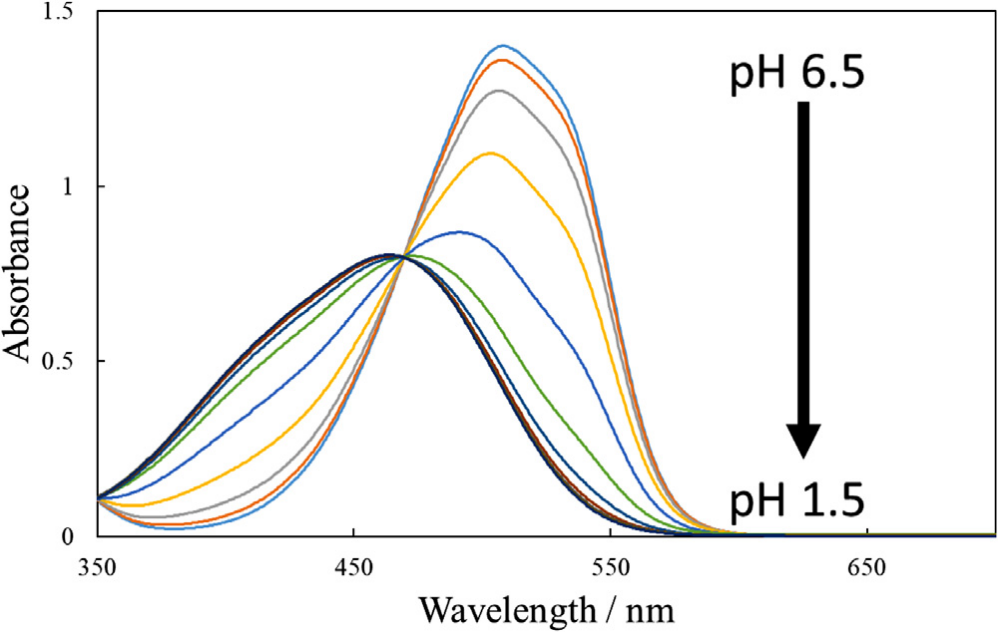
Absorption spectra of the MO solutions. The sample solutions were the same as those in Fig. 1.

**Fig. 4:**
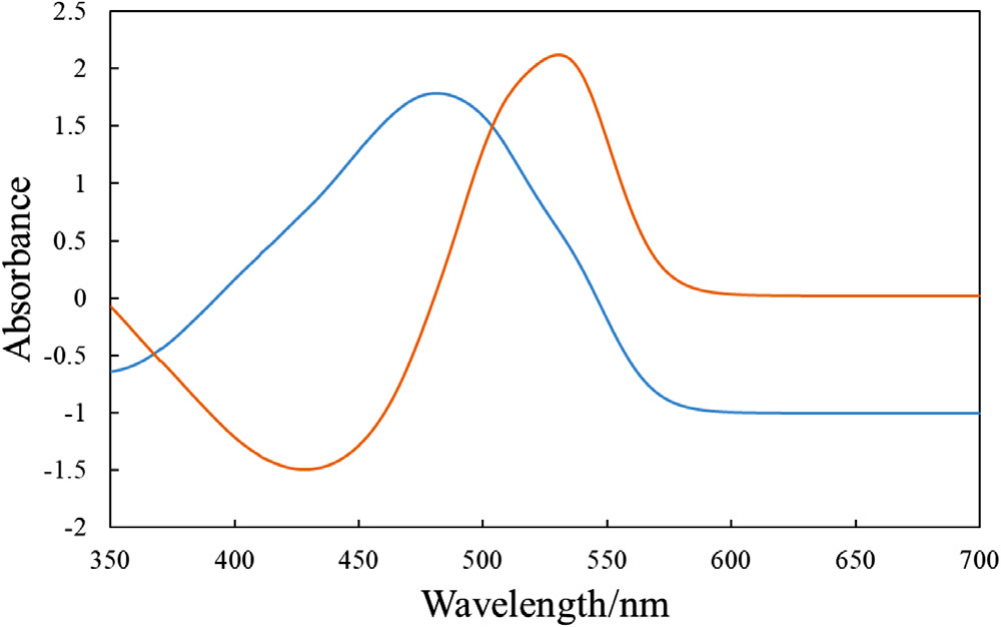
The loading spectra obtained by PCA of the spectra shown in Fig. 2.

Fig. 1 shows the microscopic transmission images of the standard MO solutions at various pH conditions. The concentration of MO was set to 1.0 × 10^−5^ mol dm^−3^. The pH values are shown in the figure. The RGB values in the region of interests (ROI) in the images were acquired by the shareware ImageJ and are summarized in Fig. 2. In this paper, three different ROIs (1022 × 822 pixels, 100 × 100 pixels and 10 × 10 pixels) were featured. Meanwhile, the absorption spectra of the standard solutions were measured with a spectrophotometer and the results are shown in Fig. 3. The loading spectra of which linear combination can express the absorption spectra of MO were obtained by least square principle component analysis (PCA) as shown in Fig. 4.

### 1.2 Calculation to reproduce absorption spectra of MO

**Fig. 5:**
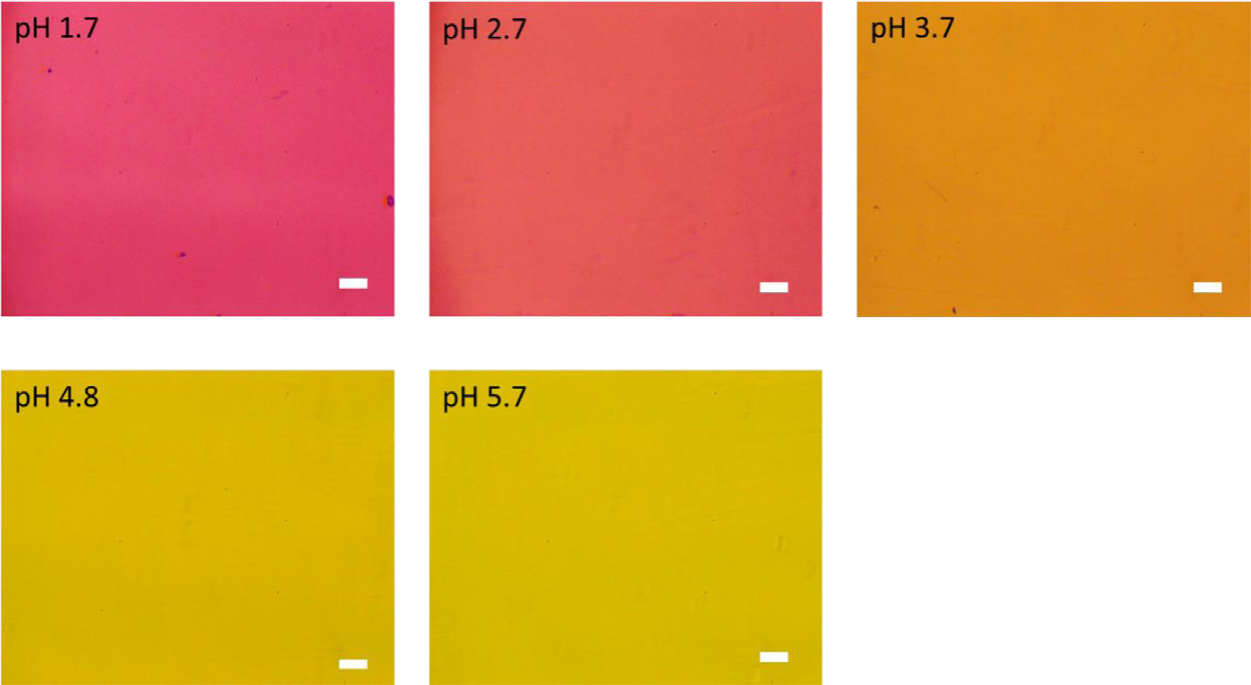
The microscopic images of aqueous MO of which absorption spectra is to be reproduced. The concentration of MO was set to 1.0 × 10^−5^ mol dm^−3^. Scale bars indicate 100 μm for every images.

**Fig. 6:**
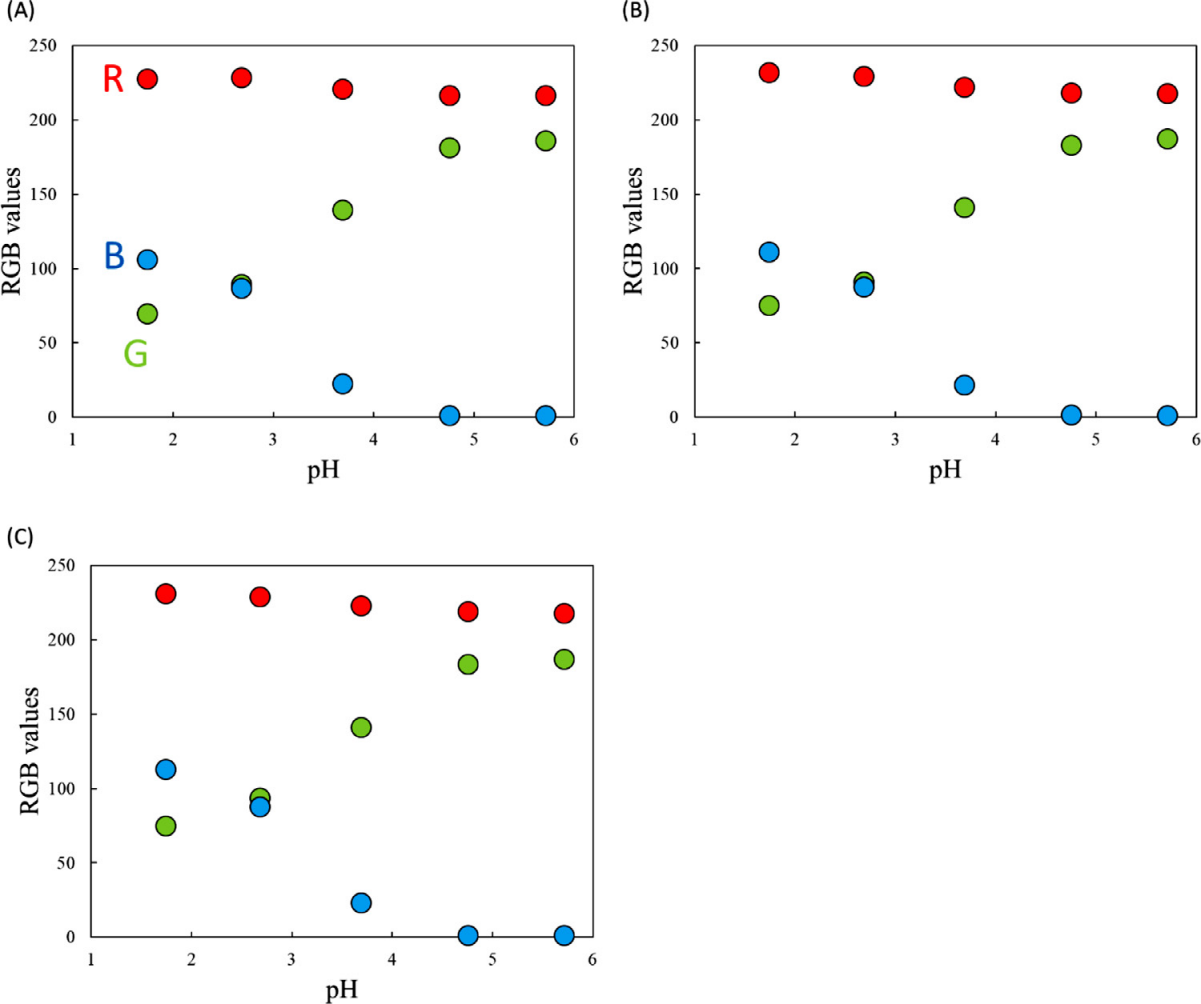
RGB values of the ROI in Fig. 5 to reproduce the absorption spectra. The ROI was set to (A)1024 × 822 pixels (B)500 × 500 pixels, and (c) 50 × 50 pixels.

**Fig. 7:**
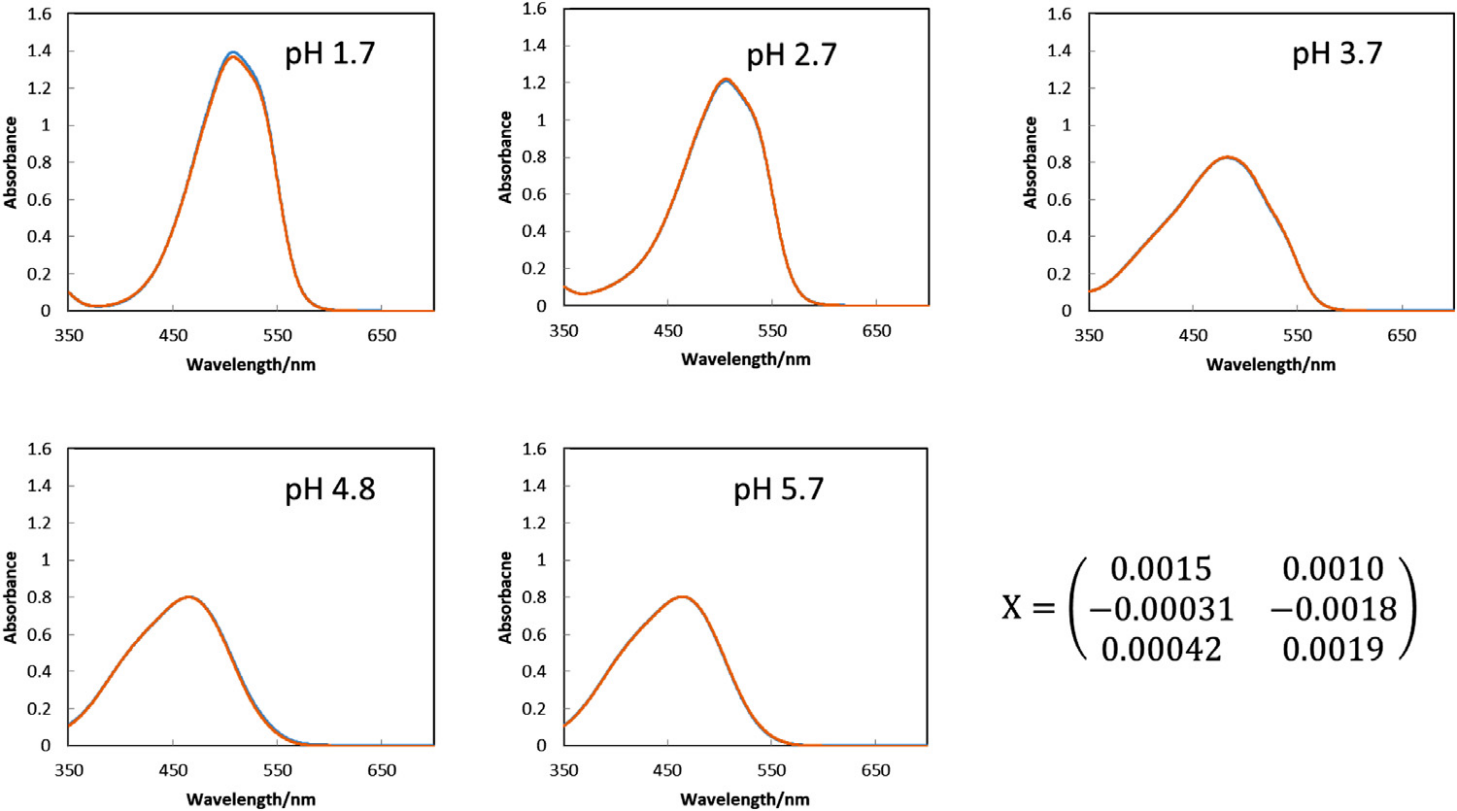
The reproduced spectra (orange line) and the spectrophotometric spectra (blue line) of the MO solutions with the ROI of 1022 × 822 pixels. The examined solution was the same as those in Fig. 5. The reproduced spectra were obtained by calculating the linear combination of the loading spectra (Fig. 4) with the score values. The score values were obtained from the RGB values in Fig. 6(A) by its conversion with the matrix shown in this figure.

**Fig. 8:**
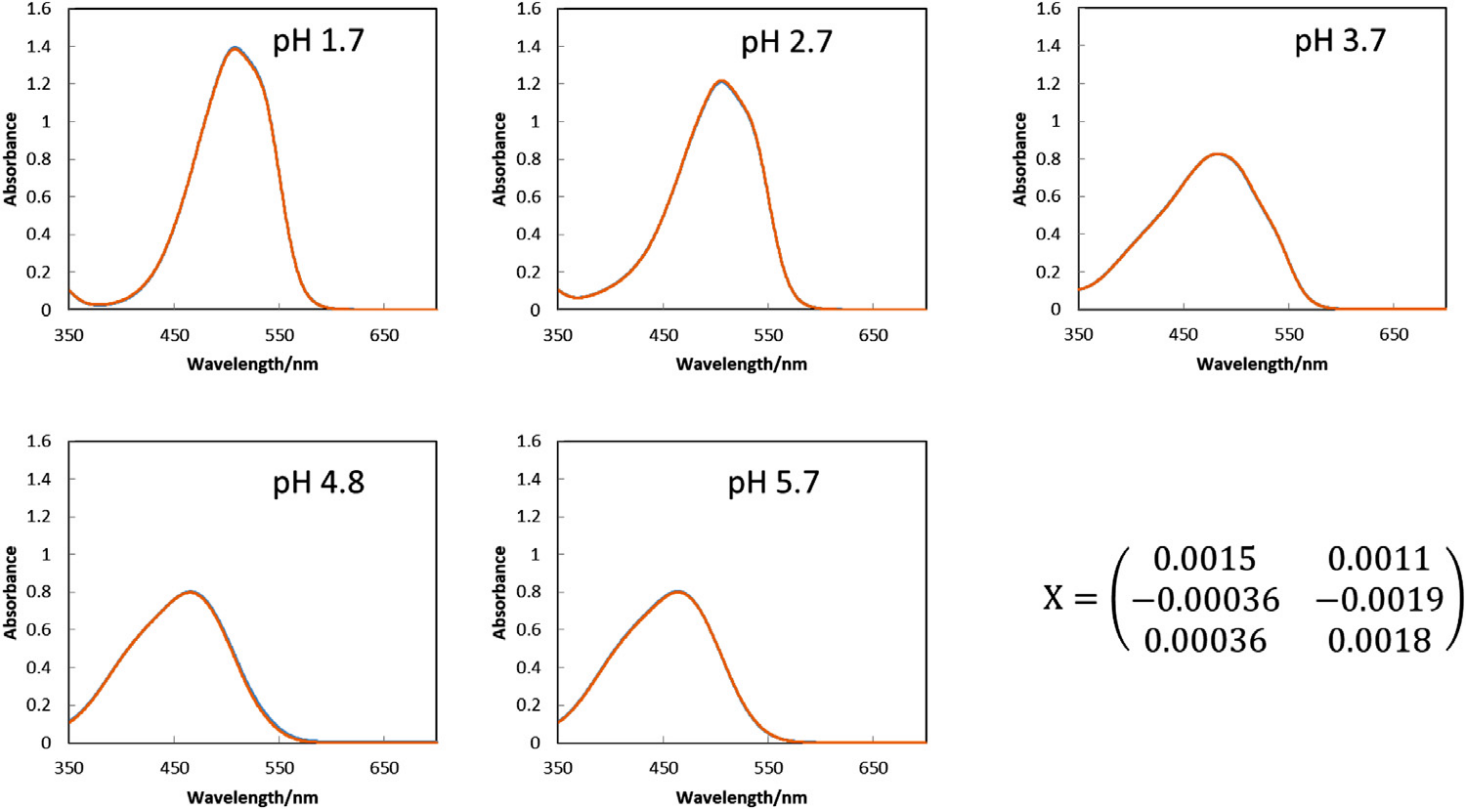
The reproduced spectra (orange line) and the spectrophotometric spectra (blue line) of the MO solutions with the ROI of 100 × 100pixels. The examined solution was the same as those in Fig. 5. The reproduced spectra were obtained by calculating the linear combination of the loading spectra (Fig. 4) with the score values. The score values were obtained from the RGB values in Fig. 6(B) by its conversion with the matrix shown in this figure.

**Fig. 9:**
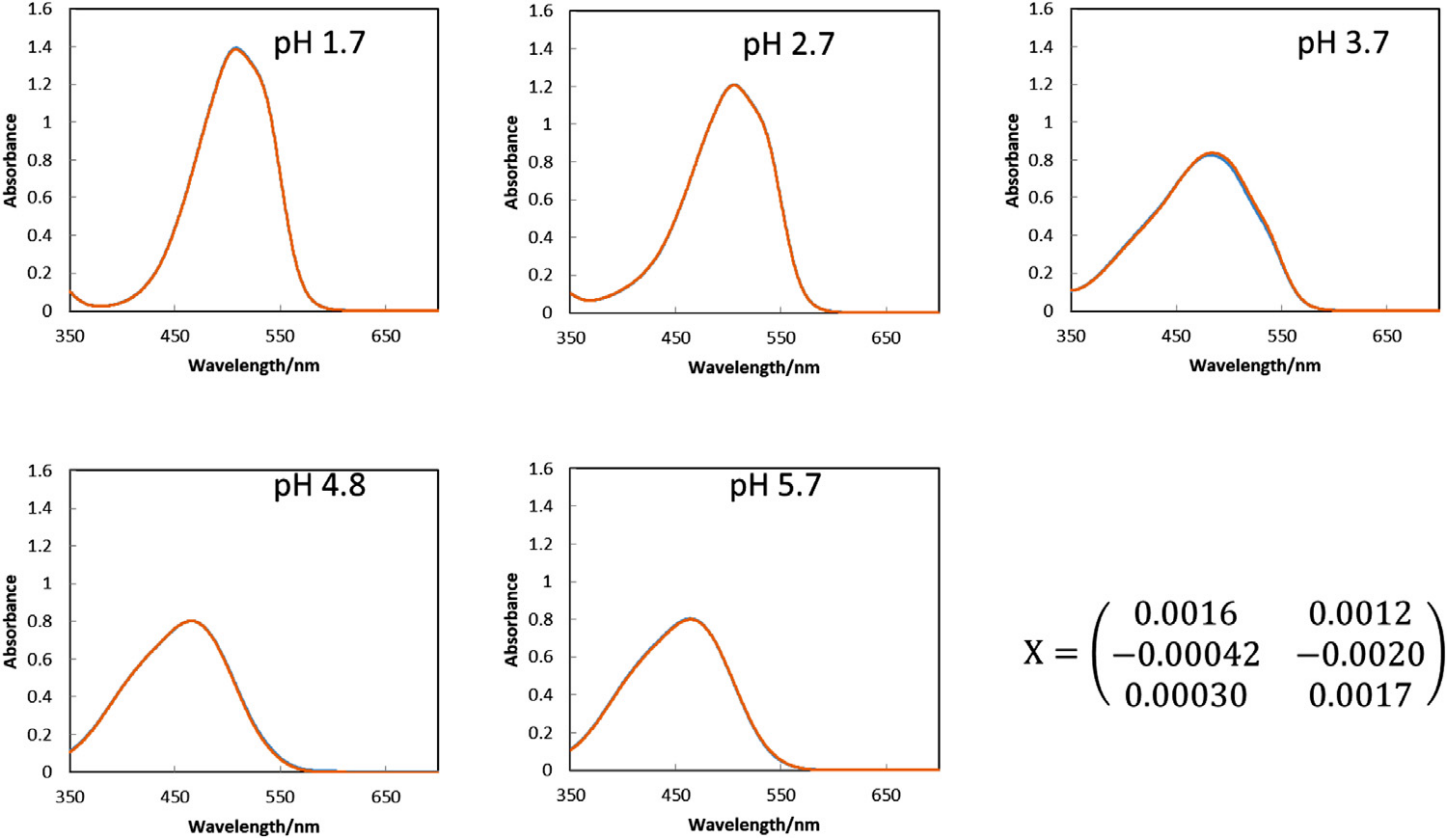
The reproduced spectra (orange line) and the spectrophotometric spectra (blue line) of the MO solutions with the ROI of 10 × 10 pixels. The examined solution was the same as those in Fig. 5. The reproduced spectra were obtained by calculating the linear combination of the loading spectra (Fig. 4) with the score values. The score values were obtained from the RGB values in Fig. 6(C) by its conversion with the matrix shown in this figure.

Fig. 5 are microscopic images of MO solutions of which absorption spectra are to be reproduced. The RGB values in the various sizes of ROI were obtained by ImageJ software as summarized in Fig. 6. The spectra reproduced by the procedure described in the experimental section with the RGB values in the ROIs of 1022 × 822 pixels, 100 × 100 pixels and 10 × 10 pixels are shown in Fig. 7, Fig. 8, Fig. 9, respectively, gathered with the spectrophotometric spectra to compare them each other. The score values for weighting the loading spectra in the linear combination were calculated by converting RGB values with the conversion matrix, **X**, shown in each figure. All the calculation procedures are conducted on Microsoft Excel 2016 sheets preserved in the supplementary material.

## 2 Experimental design, materials and methods

### 2.1 Reproducing absorption spectra of MO from RGB values of the microscopic images

Strongly note that the chemical spectra are treated as mathematical functions in this reproduction process. A matrix of chemical spectra, **A**, is expanded by least-square PCA to loading spectra, **p**_N_, and score vectors, **t**_N_. Then, the spectrum matrix can be expressed as linear combinations of these vectors as follows [2,3]:

(1) $$A=\sum t_{N}p_{N}$$

The subscript, _N_, indicates the number of the terms needed to express the spectra matrix, which is determined from eigenvalues. The loading spectra and score vectors are calculated from the absorption spectra series of the standard solutions of which pH conditions are strictly controlled. In this paper, two loading spectra are enough to fully express the MO spectra according to the eigenvalues.

Meanwhile, the microscopic images of the standard solutions are acquired by the CCD camera equipped with an inverted microscope and RGB values are obtained by image analysis. Correlation between the RGB values and the score vectors are experimentally determined by linear combination as follows [4,5]:

(2) $$\left(\begin{array}{cccc} t_{1} & t_{2} & \cdots & t_{N} \end{array}\right)=\left(\begin{array}{ccc} C_{R,\;\text{sample}} & C_{G,\;\text{sample}} & C_{B,\;\text{sample}} \end{array}\right)X$$

where **t**_N_ are the components of the score vectors, C_x,sample_ (*X*= *R*, G, B) is the RGB value of sample solutions, and **X** is defined as the conversion matrix which converts RGB values to score vectors. The conversion matrix, **X**, is determined from the absorption spectra and RGB values of standard solutions. In this case, the dimension of **X** is (3 × 2). The absorption spectra of which chemical conditions are unknown, **A**_unknown_, then can be reproduced from the RGB values of their digital images by Eq. (3) derived from Eqs. (1) and (2).

(3) $${\left(A_{\text{unknown}}\right)}^{t}=\left[\left(\begin{array}{ccc} C_{R,\;\text{sample}} & C_{G,\;\text{sample}} & C_{B,\;\text{sample}} \end{array}\right)X\right]{\left(\begin{array}{cccc} p_{1} & p_{2} & \cdots & p_{N} \end{array}\right)}^{t}+E$$

where **E** is the baseline terms, which adjust the absorbance at 700 nm to be zero.

### 2.2 Chemicals

Methyl orange (MO), methanol, sodium dihydrogen phosphate, disodium hydrogen phosphate, hydrochloric acid and sodium hydroxide were purchased from Kanto Chemical Co., Inc., Japan. All the aqueous solutions were prepared with ultrapure water purified with a PURELAB Ultra Ionic (ELGA Labwater, UK). All the chemicals were used as received.

### 2.3 Sample preparation

The stock solution of MO was prepared by dissolving MO in ethanol. The final concentration of the stock solution was set to 3.8 × 10^−3^ mol dm^−3^. Both standard and sample solutions were prepared by diluting the stock solution with aqueous solutions buffered with an acetic acid/ammonia system. The final concentration of MO was set to 1.0 × 10^−5^ mol dm^−3^. The pH values were controlled by the addition of hydrochloric acid or sodium hydroxide solutions and monitored with a pH meter (HORIBA, Japan).

### 2.4 Acquisition of microscopic images

**Fig. 10:**
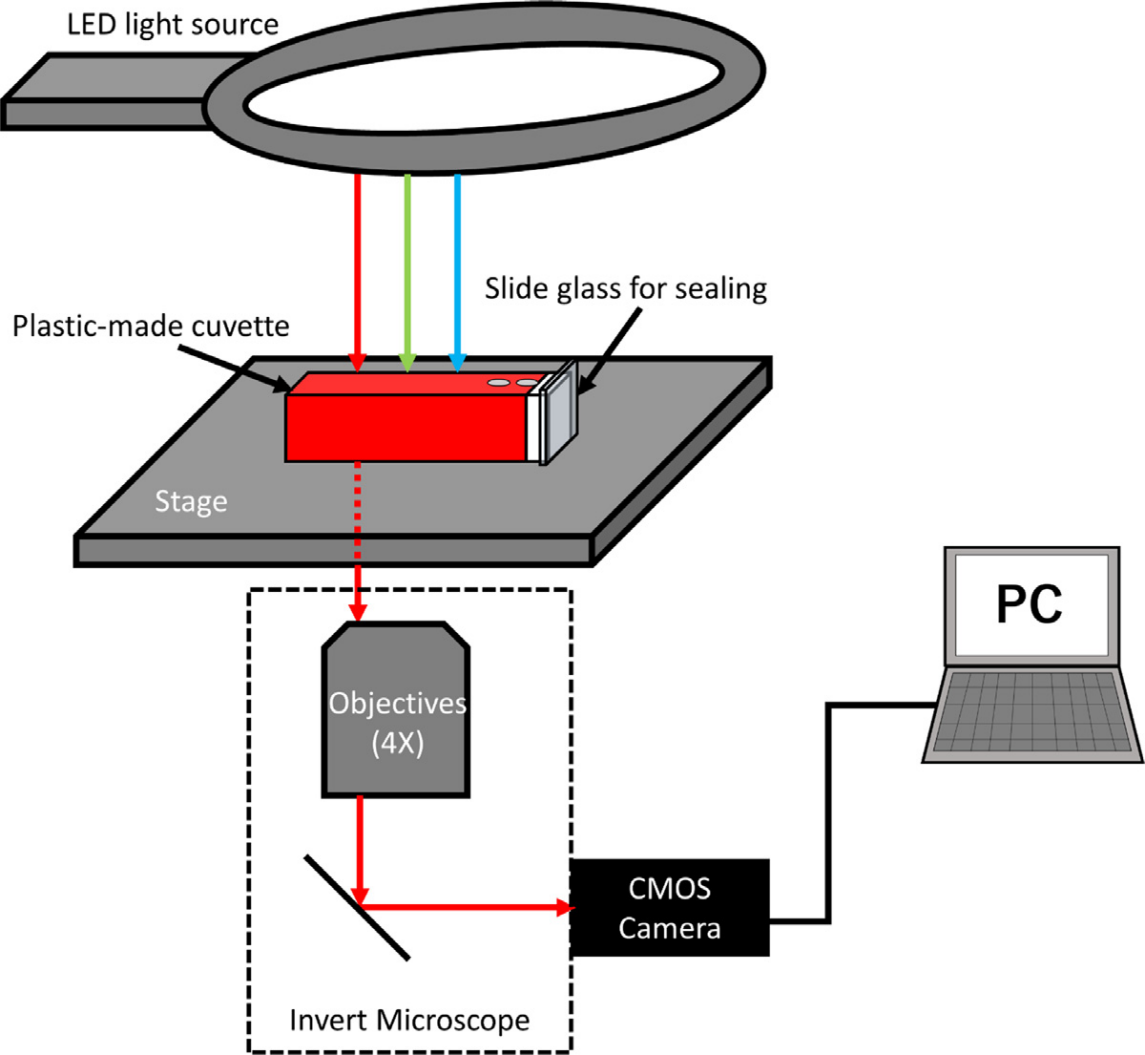
Schematic illustration of a setup for microscopic image acquisition.

A setup for the image acquisition is schematically shown in Fig. 10. Microscopic images of the solution were acquired with a CMOS color camera (Model A35140U3, OMAX Microscope, USA) equipped with an inverted microscope (Diaphot, Nikon, Japan). An objective lens (Plan 4 DL, Nikon) was used throughout the present experiment. The image acquisition process is as follows. A plastic-made cuvette (AS ONE SM-MA, optical path length 1 cm) was sealed with a slide glass, and two holes were made on the side of the cuvette for solution injection. The cuvettes were filled with the solutions and placed sideways on the stages to set 1 cm of the vertical optical path length. A white light was irradiated with an LED light source (Model MIC-209, AmScope, USA) equipped above the microscope. Prior to the acquisition of the solutions, images of pure water were taken as a reference. The light power was adjusted prior to the image acquisition so as to set the RGB values of the reference water sample to be (R, G, B) = (255,255,255).

### 2.5 Collection of absorption spectra

All the absorbance spectra were measured with a spectrophotometer (V-750, JASCO, Japan) in a plastic-made cuvette. All the spectra data were acquired with the wavelength interval of 0.5 nm.

### 2.6 Data analysis

The RGB values were obtained from the microscopic image by analysis with the shareware ImageJ (NIH, USA). The average RGB values for 100 × 100 pixels and 10 × 10 pixels were acquired at the position of (X, Y) = (309, 84), (539, 421) and (727, 208). The ROI of 1022 × 822 pixels provides the average RGB values of the whole pictures.

A series of acquired spectra of the standard solutions was analyzed by least square PCA on the freeware program KyPlot 5.0 (KyenceLab Inc., Japan) to obtain the loading spectra.

## Declaration of Competing Interest

The authors declare that they have no known competing financial interests or personal relationships which have, or could be perceived to have, influenced the work reported in this article.

## Appendix Supplementary materials

Image, application 1

Image, application 2

## References

[1] Inagawa A., Sasaki A., Uehara N. (2020). Reproducing absorption spectra of pH indicators from RGB values of microscopic images. Talanta.

[2] Beebe K.R., Pell R.J., Seasholtz M.B. (1998). Chemometrics: a Practical Guide. https://www.wiley.com/en-us/Chemometrics%3A+A+Practical+Guide-p-9780471124511.

[3] Kramer R. (1998). Chemometric Techniques for Quantitative Analysis. https://www.crcpress.com/Chemometric-Techniques-for-Quantitative-Analysis/Kramer/p/book/9780824701987.

[4] Gouzu M., Takahashi A., Tanaka N., Woo J.Y. (2009). A Method for measuring of multi spectral based omni-directional image. IPSJ SIG Tech. Rep..

[5] Nagai F., Noro Y., Takeo T., Ito H. (2012). Estimation of spectral transmission of dried seaweed from its digital image. IEEJ Trans. Electr. Electron. Eng..

