# Supplementary material for: Dataset for reproducing absorption spectra of methyl orange from the RGB values of microscopic images
Source: Data Brief. 2020 Jul 5;31:105998. doi: 10.1016/j.dib.2020.105998 (PMC7363659; doi:10.1016/j.dib.2020.105998)
Supplement: Supplementary file 2 [file mmc2.zip › 0. Description of dataset.docx]

**Description of dataset**

1. **Microscopic_Images_of_Standard_Solutions.zip**Containing microscopic images of standard solutions for determining the converting matrix, corresponding to Figure 1.
2. **Spectrophotometric_spectra_of_standard_solutions.xlsx**
   Absorption spectra of the standard solutions acquired with a spectrophotometer, corresponding to Figure 2.
3. **RGB_values_of_standard_solutions.xlsx**RGB values of the regions of interests (ROIs) in microscopic images stored in “**Microscopic_Images_of_Standard_Solutions.zip**”, corresponding to Figure 3. Values were obtained by analyzing with ImageJ software.
4. **MO_PCA_Results.kyp**Least-square principle component analysis of a series of absorption spectra of the standard solutions, corresponding to Figure 4. The file can be opened with a shareware Kyplot, which can be download here for free; <https://www.kyenslab.com/en-us/download-en/>.
5. **Microscopic_Images_of_Sample_Solutions.zip**
   Containing microscopic images of sample solutions whose absorption spectra are to be reproduced, corresponding to Figure 5.
6. **RGB_values_of_sample_solutions.xlsx**RGB values of the ROIs in the microscopic images stored in “**Microscopic_Images_of_Sample_Solutions.zip**”, corresponding to Figure 6. Values were obtained by analyzing with ImageJ software.
7. **Calculation_sheet_for_reproducing_absorption_spectra.zip**
   Microsoft Excel sheet for reproducing absorption spectra with the loading spectra and score values converted from the RGB values, corresponding to Figures 7 (1022_822_Reproduction_Calculation_Sheet), 8 (100_100_Reproduction_Calculation_Sheet) and 9 (10_10_Reproduction_Calculation_Sheet).
8. **Spectrophotometric_spectra_of_sample_solitions.xlsx**Absorption spectra of the sample solutions acquired with a spectrophotometer, corresponding to Figure 7, 8, 9(orange lines).
